# Ficolin-2 and ficolin-3 in women with malignant and benign ovarian tumours

**DOI:** 10.1007/s00262-013-1445-3

**Published:** 2013-06-07

**Authors:** Agnieszka Szala, Sambor Sawicki, Anna St. Swierzko, Janusz Szemraj, Marcin Sniadecki, Mateusz Michalski, Andrzej Kaluzynski, Jolanta Lukasiewicz, Anna Maciejewska, Dariusz Wydra, David C. Kilpatrick, Misao Matsushita, Maciej Cedzynski

**Affiliations:** 1 Laboratory of Immunobiology of Infections, Institute of Medical Biology, Polish Academy of Sciences, Lodowa 106, 93-232 Lodz, Poland; 2 Chair and Department of Gynaecology, Oncologic Gynaecology and Gynaecologic Endocrinology, Medical University of Gdansk, Kliniczna 1a, 80-402 Gdańsk, Poland; 3 Department of Biochemistry, Medical University of Lodz, Mazowiecka 6/8, 92-215 Lodz, Poland; 4 Institute of Microbiology, Immunology and Biotechnology, University of Lodz, Banacha 12/16, 90-237 Lodz, Poland; 5 Department of Clinical Pathomorphology, Polish Mother`s Memorial Hospital Research Institute, Rzgowska 281/289, 93-338 Lodz, Poland; 6 Department of Immunochemistry, Ludwik Hirszfeld Institute of Immunology and Experimental Therapy, Polish Academy of Sciences, Weigla 12, 53-114 Wroclaw, Poland; 7 Scottish National Blood Transfusion Service, National Science Laboratory, Ellen’s Glen Road, Edinburgh, EH17 7QT Scotland UK; 8 Department of Applied Biochemistry, Tokai University, 4-1-1 Kitakaname, Hiratsuka-shi, Kanagawa 259-1292 Japan

**Keywords:** Ovarian cancer, Benign ovarian tumours, Ficolin-3 (H-ficolin), Ficolin-2 (L-ficolin), *FCN2* gene, *FCN3* gene

## Abstract

Ficolins are serum pattern recognition molecules. They have opsonic properties and are able to activate complement via the lectin pathway. This paper reports investigations concerning ficolin-2 and ficolin-3 in ovarian cancer (OC). Their serum levels, single nucleotide polymorphisms of the corresponding *FCN2* and *FCN3* genes and specific mRNA expression in ovarian sections were investigated in 128 patients suffering from primary OC and 197 controls operated on for reasons other than malignancies. The latter consisted of two reference groups: those with benign tumours (*n* = 123) and those with normal ovaries (NO) (*n* = 74). Serum ficolin-2 and ficolin-3 concentrations were higher among patients with malignant disease when compared with either of the reference groups. A significant correlation between ficolin-2 and ficolin-3 concentrations was found, while no correlations with CA125 antigen or CRP were observed. No differences in the frequency of single nucleotide polymorphisms at sites −64, −4 (promoter), +6359, or +6424 (exon 8) (*FCN2* gene) nor in the frame-shift mutation *1637delC* (*FCN3* gene) were found between investigated groups. In contrast to serum concentrations, the expression of *FCN2* gene (reported for the first time in ovarian sections) was significantly lower in women with OC in comparison with patients with NO but not with benign ovarian tumours. In case of *FCN3* gene, its expression levels in OC group inversely correlated with serum ficolin-3 and were lower in comparison with controls.

**Electronic supplementary material:**

The online version of this article (doi:10.1007/s00262-013-1445-3) contains supplementary material, which is available to authorized users.

## Introduction

Ficolins constitute a group of major serum pattern recognition molecules, structurally and functionally related to collectins. They are able to opsonize infecting microorganisms and to activate the lectin pathway of complement in cooperation with mannan-binding lectin (MBL)-associated serine proteases (MASPs). Ficolin-1 (M-ficolin) is predominantly a cellular molecule associated with monocytes and neutrophils; ficolin-2 (L-ficolin) and ficolin-3 (H-ficolin) are predominantly serum molecules [1]. The distinguishing feature of ficolin-2 is a unique set of binding sites arranged within an internal cleft enabling it to recognize a relatively wide variety of ligands. Among its microbial target structures, there are bacterial capsular polysaccharides, lipoteichoic acids and 1,3-β-glucans of fungal origin (reviewed by Kilpatrick and Chalmers [1]). The knowledge concerning microbial specificity of ficolin-3 is limited. It was shown to interact with *Aerococcus viridans* polysaccharide and lipopolysaccharides of some *Hafnia alvei* strains [2, 3]. Both ficolin-2 and ficolin-3 are, however, able to recognize certain endogenous ligands, like apoptotic cells. Thus, ficolins may contribute to their clearance and, in consequence, maintain tissue homeostasis [4, 5].

Ficolin-2 is synthesized in the liver by the *FCN2* gene located on chromosome 9. Several single nucleotide polymorphisms (SNPs) affecting its concentration and/or activity have been reported. Among them, the combination of −64 A>C (rs78654553, promoter region) and +6424 G>T (rs7851696, exon 8) SNPs, found in strong linkage disequilibrium, is associated with low L-ficolin levels in carriers of minority alleles. Another pair, −4 A>G (rs17514136, promoter) and +6359 C>T (rs17549193, exon 8), has the opposite effect [6–9]. However, no case of total ficolin-2 deficiency has been described to date.

Ficolin-3 is synthesized mainly in the liver and lung and was suggested to participate in both systemic and local innate immune responses [7, 10]. A frameshift mutation of the *FCN3* gene (located on chromosome 1), *1637delC,* leads to the total deficiency in variant homozygotes [11, 12]. Three cases of such a deficiency and associated symptoms have been described [12–14]. Moreover, it has been suggested that H-ficolin may be a potential indicator of ovarian cancer (OC) [15].

Extra-hepatic expression of ficolin genes has previously been described in the bone marrow, tonsils, intestine and foetal lung (*FCN2*) or heart, spleen, kidney, pancreas, brain and placenta (*FCN3*) [16]. Moreover, Kuraya et al. [17] found a significant ficolin-3 expression in T98G glioma cell line. So far, no data concerning expression of ficolins in the ovary are available; however, certain collectins (mannan-binding lectin, surfactant protein D, collectin-11) and MASP-2-specific mRNA or proteins have been detected in this organ [18–20].

We report for the first time the expression of the *FCN2* and *FCN3* genes in the ovary, their polymorphisms, and their serum concentrations in patients with ovarian tumours (both malignant and benign) as well as women operated on for reasons unconnected with ovarian tumours.

## Materials and methods

### Patients and controls

Whole blood, serum and/or ovarian tissue samples were obtained from 325 patients from the Department of Gynaecology, Oncologic Gynaecology and Gynaecologic Endocrinology, Medical University of Gdansk, Poland. A total of 128 patients (aged 28–86 years, mean 58.6) had the diagnosis of primary ovarian cancer (OC group). The majority of patients suffered from serous carcinoma (*n* = 83). Twenty-five patients had ovarian tumours of other histological types, including endometrioid, mucinous, clear-cell carcinomas. According to FIGO staging, 78 patients had advanced disease stage (FIGO III–IV), while 25 were classified to FIGO I–II. Sixty-five women had poorly differentiated tumours (G3), while in 33 cases, the histological differentiation was defined as G1-2. For some patients, complete clinical data were not available. Two groups of women undergoing surgery for reasons other than malignancies were collectively classified as controls (C): 123 were diagnosed with benign tumours of the ovary (BT; aged 19–82 years, mean 45.6), while 74 were operated on because of leiomyomas or dysfunctional uterine bleeding but had no true pathological changes in the ovaries (NO group; aged 20–76 years, mean 48.2). The BT group included patients with ovarian serous cysts, adenomas, fibromas, endometriosis and teratomas. Blood from all patients was taken pre-operatively while tissue samples—during primary surgery. Additionally, some clinical material collected previously from OC patients [18] was investigated: sera taken before and after surgery. Approval of the local ethical committee was obtained, as was written informed consent of patients.

### Blood, serum and ovarian tissue samples

Blood samples for DNA preparation were taken into tubes containing sodium citrate and stored at −20 °C. Sera were prepared from blood samples collected into tubes without anticoagulant and stored at −70 °C until testing. Tissue samples were collected into tubes with RNAlater (Life Technologies, USA) and stored at −70 °C.

### Determination of ficolin-2 and ficolin-3 concentrations

Serum ficolin-2 and ficolin-3 concentrations were measured by ELISA as described previously by Kilpatrick et al. [21] and Michalski et al. [14], respectively. Relative insufficiency (low concentrations) was taken as <0.8 μg/ml (ficolin-2) or <10 μg/ml (ficolin-3), while high levels were taken as >2.9 μg/ml (ficolin-2) or >18.5 μg/ml (ficolin-3). Low values corresponded to 10th percentile and high values to 75th percentile among controls. As there are no standard values, they were chosen arbitrarily.

### Investigation of single nucleotide polymorphisms of the *FCN2* and *FCN3* genes

DNA was extracted from blood samples with the use of GeneMATRIX Quick Blood Purification Kit (EURx Ltd, Poland), according to the manufacturer’s protocol. Single nucleotide polymorphisms of the *FCN2* gene were analysed as recently described by Szala et al. [22]. Briefly, polymorphisms at positions −64 (A>C), +6359 (C>T) and +6424 (G>T) were investigated with the use of PCR employing allele-specific primers. For an investigation of −4 A>G polymorphism, a PCR–RFLP protocol was used, employing MboII endonuclease. The sequences of primers used for genotyping are presented in the supplementary material (Tables S1–S4). The presence of *FCN3* gene *1637delC* polymorphism was investigated by a PCR–RFLP method, essentially as described previously [14]. The sequences of primers are shown in supplementary Table S5.

### Detection of *FCN2* and *FCN3* gene expression using real-time PCR method

The *FCN2* and *FCN3* gene expression levels were investigated as previously described by Swierzko et al. [18] for the mannan-binding lectin (*MBL2*) and MASP-2 (*MASP2*) genes. PCR primers for *FCN3* came from Qiagen (USA), while PCR primers for *FCN2* had sequences published by Kuraya et al. [17]. Primers specific for TATA-box protein (TBP, used as a housekeeping gene) had sequences described by Li et al. [23].

Samples were incubated at 50 °C for 2 min and at 95 °C for 10 min and then subjected to 40 cycles of 95 °C for 30 s, 56 °C for 1 min and 72 °C for 1 min. SYBR Green I fluorescence emission data were captured, and mRNA levels were quantified using the critical threshold (C_t_) value. Analyses were performed with ABI Prism 7700 (SDS software). Results were normalized to values obtained for TBP. Relative gene expression levels were obtained using the ∆∆C_t_ method [24]. Specificity of amplification was further confirmed by obtaining melting curve profiles.

### Statistical analysis

The Statistica (version 10, StatSoft Poland) software package was used for data management and statistical calculations. Median serum L-ficolin and H-ficolin concentrations as well as *FCN2* and *FCN3* gene expressions in ovarian sections were compared by the Mann–Whitney *U*-test. Correlations were determined by Spearman’s test. The frequencies of *FCN2* and *FCN3* gene variants were compared by Fisher’s exact test (2-sided). Pre- and post-operative protein levels were compared with the help of Wilcoxon’s test. *p* values <0.05 were considered statistically significant.

## Results

### Ficolin-2 and ficolin-3 concentrations in sera

**Fig. 1:**
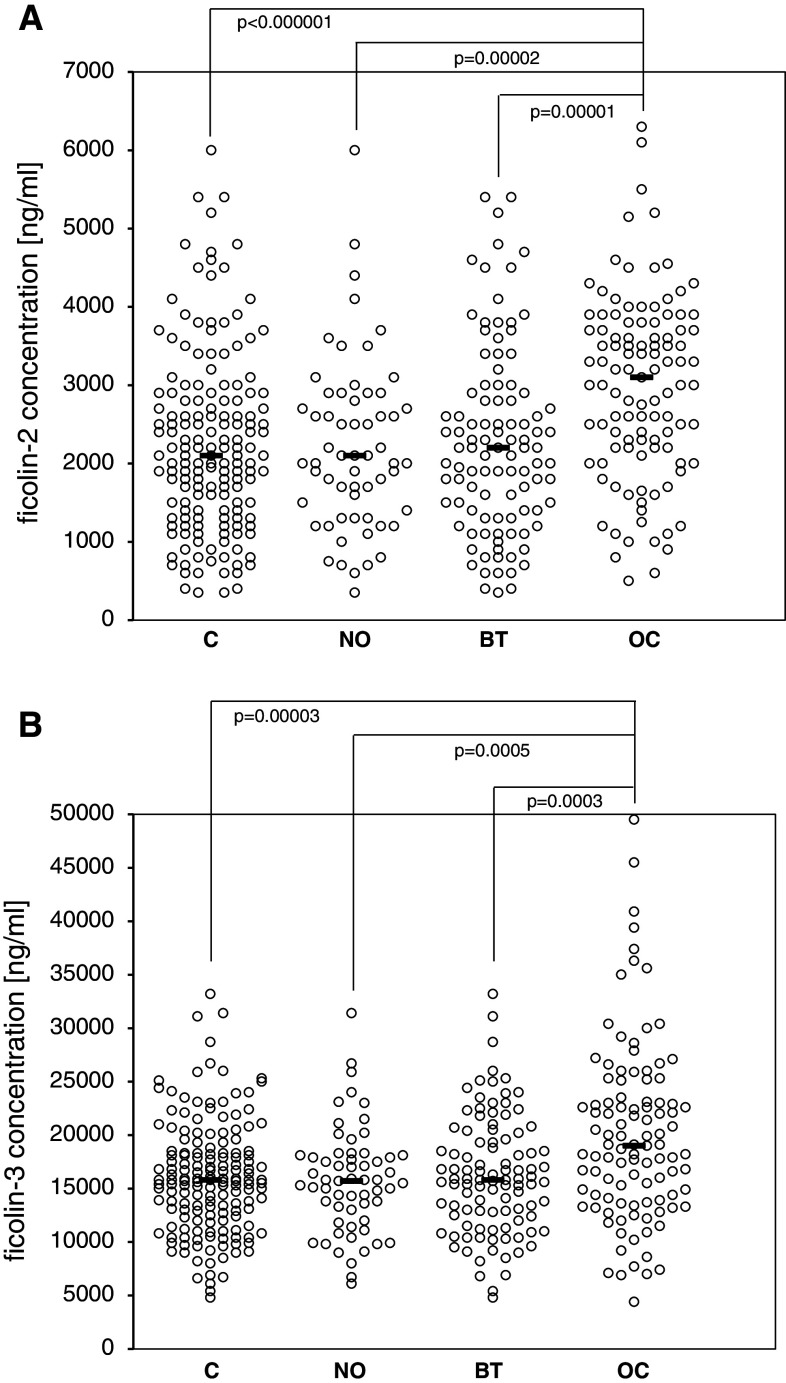
Individual ficolin-2 (**a**) and ficolin-3 (**b**) concentrations in sera of patients. C—control group; NO—women with normal ovaries; BT—patients with benign ovarian tumours; OC—patients with primary OC. *Bars* indicate median values

The median pre-operative serum ficolin-2 concentration in the control group (C) was 2.1 μg/ml. Average levels for NO and BT subgroups were similar (2.1 and 2.2 μg/ml, respectively). However, increased serum ficolin-2 was associated with the cancer patients (median 3.1 μg/ml; *p* < 0.000001 compared with C group) (Fig. 1a). Consequently, high serum ficolin-2 (>2.9 μg/ml) concentrations were more frequent in cancer patients (Table 1a). In contrast, no differences were found in the frequency of low (<0.8 μg/ml) levels between any of the reference and OC groups. Determination of serum ficolin-2 level had a specificity of 78.4 % and sensitivity of 53.2 % for OC. Positive predictive equalled 62.8 %, while negative predictive value was 71 %.

**Table 1:** (a) Frequency (%) of low (<0.8 μg/ml) and high (>2.9 μg/ml) ficolin-2 serum concentrations; (b) frequency (%) of low (<10 μg/ml) and high (>18.5 μg/ml) ficolin-3 serum concentrations

Group	Low ficolin-2 concentrations	High ficolin-2 concentrations	Statistical significance*	Odds ratio (95 % CI)*
(*a*)				
C	8	21.6		
NO	8.3	18.3		
BT	8	23.5	*p* = 0.55^b^	1.37 (0.62–3.04); *p* = 0.44^b^
OC	1.9	55.1	*p* = 0.0001^a^ *p* = 0.0007^b^ *p* = 0.001^c^	4.46 (2.61–7.61); *p* < 0.0001^a^ 5.48 (2.57–11.67); *p* < 0.0001^b^ 3.99 (2.2–7.25); *p* < 0.0001^c^

Group	Low ficolin-3 concentrations	High ficolin-3 concentrations	Statistical significance*	Odds ratio (95 % CI)*
(*b*)				
C	12.3	24.7		
NO	14.8	18		
BT	10.9	28.7	*p* = 0.14^b^	1.83 (0.83–4); *p* = 0.13^b^
OC	7.5	51.9	*p* < 0.000001^a^ *p* < 0.000001^b^ *p* = 0.001^c^	3.29 (1.95–5.54); *p* < 0.0001^a^ 4.9 (2.3–10.44); *p* < 0.0001^b^ 2.68 (1.57–4.76); *p* = 0.0008^c^

* Data for high levels shown only

^a^C; ^b^ NO; ^c^ BT

The median ficolin-3 level among controls was 15.8 μg/ml (15.7 μg/ml and 15.8 μg/ml within NO and BT subgroups, respectively). That was significantly lower than in OC patients (19 μg/ml; *p* = 0.00003) (Fig. 1b). As with ficolin-2, high ficolin-3 levels (>18.5 μg/ml) were significantly commoner in OC than in the reference groups (Table 1b), while the incidence of low ficolin-3 concentrations (<10 μg/ml) did not differ between these groups. Determination of ficolin-3 concentration had a specificity of 75.3 % and sensitivity of 51.9 % for OC. Positive predictive value was 57.9 %, while negative predictive value was 70.5 %.

No significant difference in serum level of either ficolin was found when patients classified into OC subgroups, depending on histological type of tumour, its differentiation or disease stage, were compared (not shown).

In both C and OC groups, serum levels of ficolin-2 and ficolin-3 correlated significantly (*R* = 0.217; *p* = 0.006 and *R* = 0.347; *p* = 0.0003, respectively; supplementary material, Fig. S1A, B). Moreover, ficolin-3 concentrations correlated with age (C: *R* = 0.285, *p* = 0.0002; OC: *R* = 0.317, *p* = 0.0009), but within the control group, the highest median concentration was found in women aged 55–64 years (18.2 μg/ml). No correlation with age was found in the case of ficolin-2. Neither ficolin-2 nor ficolin-3 levels correlated with CA-125 or CRP. Median serum concentrations of both lectins determined 24–48 h after cytoreduction were significantly lower than before surgery (ficolin-2: *p* = 0.02; ficolin-3: *p* = 0.004) (supplementary material, Fig. S2A, B). A highly significant correlation between pre- and post-surgery values was observed (*R* = 0.832, *p* = 0.0008 and *R* = 0.847; *p* = 0.00002, respectively).

### Polymorphisms of *FCN2* and *FCN3* genes

*FCN2* gene polymorphisms at positions: −64; −4; +6359; +6424 were analysed in 175 control women (68 NO, 107 BT) and 118 patients suffering from primary OC. Full genotypes (each of four sites) were determined successfully in 171 controls and 114 OC patients. No significant differences in frequency of genotypes (Table 2) or alleles (not shown) were found between cancer patients and controls. Within the control group, the commonest full genotype was A/A–A/G–C/T–G/G (*n* = 50; 29.2 %), followed by A/A–A/A–C/C–G/G (*n* = 44; 25.7 %) and A/A–G/G–T/T–G/G (*n* = 19; 11.1 %). Among OC patients, the most frequent genotype (A/A–A/A–C/C–G/G) was found in 39 cases (34.5 %), while A/A–A/G–C/T–G/G and A/A–G/G–T/T–G/G were found in 27 (23.7 %) and 11 (9.6 %), respectively.

**Table 2:** Frequency of *FCN2* genotypes in OC patients and controls

Polymorphism/genotype frequency	Group			
	C	NO	BT	OC
−64 A>C				
A/A	78.7	74.6	81.3	77.8
A/C	20.1	23.9	17.8	18.0
C/C	1.1	1.5	0.9	4.3
−4 A>G				
A/A	46.0	44.1	47.2	52.5
A/G	42.5	44.1	41.5	37.2
G/G	11.5	11.8	11.3	10.2
+6359 C>T				
C/C	41.6	43.3	40.6	47.4
C/T	43.9	43.3	44.3	41.4
T/T	14.5	13.4	15.1	11.2
+6424 G>T				
G/G	78.9	73.5	82.2	74.6
G/T	19.4	25.0	15.9	20.3
T/T	1.7	1.5	1.9	5.1

As expected, serum ficolin-2 levels within groups depended on genotype. For example, among control wild-type homozygotes for −64 A>C and +6424 G/T polymorphism (A/A–X–X–G/G, where “X” corresponds to any variant), median L-ficolin concentration was 2.25 μg/ml, while among carriers of other genotypes (M–X–X–M, where “M” signifies the presence of at least one mutated allele), the median was 1.3 μg/ml (*p* = 0.006). In contrast, control wild-type homozygotes for −4 A>G and +6359 C>T (X–A/A–C/C–X) had significantly lower serum L-ficolin (1.8 μg/ml) than their counterparts (X–M–M–X) (2.2 μg/ml; *p* = 0.02).

Ficolin-2 concentrations among patients and controls carrying similar genotypes were compared (analyses were performed when the number in each group exceeded 15). Significantly higher ficolin-2 levels were found in OC patients than in any of the reference groups (C, NO, BT) for all five variants (Table 3).

**Table 3:** Ficolin-2 concentrations in OC patients and controls, according to *FCN2* genotype

Genotype	Group			
	C	NO	BT	OC
A/A–A/A–C/C–G/G	2.4 (41) *p* = 0.002	2.1 (16) *p* = 0.03 *p* = 0.9^#^	2.4 (25) *p* = 0.006	3.0 (38)
A/A–M–M–G/G^a^	2.2 (69) *p* = 0.001	2.1 (25) *p* = 0.002 *p* = 0.6^#^	2.2 (44) *p* = 0.007	3.4 (35)
A/A–X–X–G/G^b^	2.25 (122) *p* = 0.00002	2.0 (44) *p* = 0.0001 *p* = 0.41^#^	2.3 (78) *p* = 0.0003	3.3 (78)
X–A/A–C/C–X^b^	1.8 (63) *p* = 0.000003	1.85 (24) *p* = 0.002 *p* = 0.42^#^	1.8 (39) *p* = 0.000009	3.0 (49)
X–M–M–X^a^	2.2 (84) *p* = 0.0009	2.1 (32) *p* = 0.002 *p* = 0.69^#^	2.2 (52) *p* = 0.008	3.3 (49)

Numbers of cases given in parentheses; *p* values versus OC or BT (^#^) group

^a^–M—at least one variant (minority) allele present (genotypes: A/G or G/G for SNP at position −4 and C/T or T/T for SNP at position +6359

^b^–X—any genotype

The *FCN3* gene *1637delC* frame-shift mutation was investigated in 114 OC patients and 167 controls. None of the subjects was homozygous for the deletion (−/− genotype). The number of C/− heterozygotes in the OC group did not differ significantly from that in any of reference groups (Table 4). Although ficolin-3 levels were determined in only 6 control and 4 OC heterozygotes, it is evident that they are much lower than among C/C genotype subjects (*p* = 0.00004, supplementary material, Fig. S3). However, median ficolin-3 level among C/− OC patients is twice as high as that of corresponding controls (13.6 μg/ml vs. 6.8 μg/ml). Consequently, the concentrations differed significantly between OC and control C/C homozygotes (19.1 μg/ml vs. 15.8 μg/ml; *p* = 0.00004).

**Table 4:** Frequency (%) of *1637delC* mutation (*FCN3*) among OC patients and controls

Group/subgroup	% of C/− heterozygotes	delC (−) allele frequency
C	3.6	0.018
NO	6.2	0.031
BT	2	0.01
OC	3.5	0.018

### Ficolin-2 and ficolin-3 specific mRNA expression in ovarian sections

**Fig. 2:**
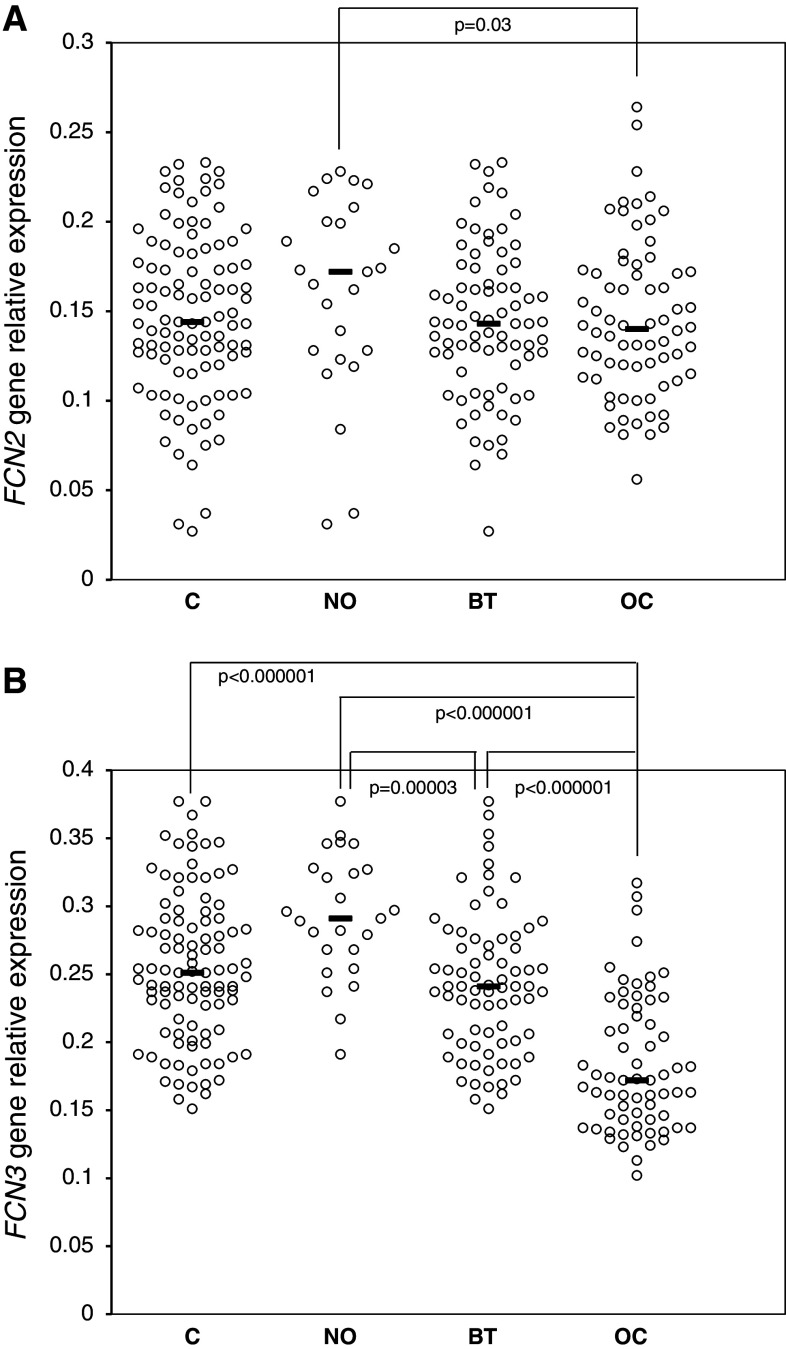
Expression (at the mRNA level) of *FCN2* (**a**) and *FCN3* (**b**) genes in ovarian section samples. C—control group; NO—women with normal ovaries; BT—patients with benign ovarian tumours; OC—patients with primary ovarian cancer. *Bars* indicate median values

The expression of both *FCN2* and *FCN3* genes, at the mRNA level, was detected in both OC patients and controls. *FCN2* gene relative expression was determined in ovarian sections from 103 controls (25 NO and 78 BT), and 64 patients suffering from primary OC. Although no significant difference between these groups was observed (Fig. 2a), there was a clear difference between OC patients and the NO group: the median relative expression in the latter was 1.26-fold higher than in the former (*p* = 0.03). No significant differences were found when patients classified into OC subgroups, depending on histological type of tumour, its differentiation or disease stage were compared (not shown).

The real-time quantitative PCR for detection of the *FCN3* gene expression was performed on ovarian sections from 101 women classified as controls (25 NO and 76 BT) and 72 subjects with primary OC (Fig. 2b). The median expression level was significantly lower in OC than in the control group and notably in the NO subgroup (1.46-fold and 1.69-fold difference, respectively; *p* < 0.000001). Both control subgroups exhibited higher expression relative to the OC group, but the BT subgroup had significantly lower expression compared with the NO subgroup (*p* = 0.00003). Data from OC subgroups, depending on histological type of tumour, differentiation grade or disease stage, were similar (not shown). Moreover, an inverse correlation between *FCN3* gene expression and ficolin-3 levels among OC patients was observed (*R* = −0.283; *p* = 0.03, supplementary material, Fig. S4).

Gene expression levels did not correlate with age of patients, CA125 antigen or CRP concentrations in any of the groups or subgroups analysed (not shown). However, a correlation between *FCN2* and *FCN3* gene expression was found in the control group (*R* = 0.246; *p* = 0.01). There was no correlation between *FCN2* and *FCN3* expression in the OC group.

## Discussion

Many inflammatory factors have been shown to be up-regulated in sera of patients suffering from malignancy. Bertenshaw et al. [25] suggested that although inflammatory reactions are involved in numerous diseases of various etiologies, the relative ratio or expression patterns of inflammatory molecules may be disease-specific. Previously, we demonstrated higher MBL levels and higher MBL-MASP-2 complex activity in patients suffering from primary OC (*MBL2* A/A genotype) compared with healthy controls [18]. Similarly, elevated MBL and MASP-2 concentrations and activities were found in patients with colon cancer [26, 27]. However, there are few previous studies relating ficolins to cancer, and the entire literature can be summarized as follows. Vazquez-Ortiz et al. [28] found over-expression of the *FCN2* gene in human papillomavirus-positive cervical cancer. Later, Arellano-Garcia et al. [29] considered ficolin-2 as a potential biomarker in metastatic oral cancer. Kilpatrick et al. [30] and Ameye et al. [31] reported that low ficolin-2 or ficolin-3 do not influence the risk of chemotherapy-related infections in haematological malignancies. However, low ficolin-1 (M-ficolin) serum levels were associated with severe infections in patients [31]. The *FCN3* gene was found to be under-expressed in hepatocellular and squamous cell lung carcinomas [32, 33]. Schlapbach et al. [34] reported higher ficolin-3 serum concentrations in paediatric patients with acute leukaemia than in children with other malignancies. Andersen et al. [15], using differential in-gel electrophoresis (DIGE), found higher amount of ficolin-3 in sera of OC patients relative to controls and suggested ficolin-3 could be a useful biomarker in that malignancy.

In this study, we have confirmed the elevation of ficolin-3 in OC and obtained similar results with ficolin-2 (Fig. 1). The serum levels of both ficolins in OC patients were higher than in controls carrying the same *FCN2* or *FCN3* genotypes. The sensitivity, specificity and positive predictive value of ficolin-2 and ficolin-3 levels were similar to these reported previously for C-reactive protein (CRP) [35]. We did not find correlation between ficolins and CRP or CA125. It is noteworthy that others have found correlation between ficolin-3 and CRP in childhood haematological malignancies [34]. It should be stressed, that elevated levels of ficolins have also been reported in other, non-cancerous diseases [36–39].

In contrast to high serum levels, low local expression of the *FCN2* and *FCN3* genes seems to be associated with OC (Fig. 2). Relative expression values for both ficolins differed most obviously between the OC and the NO groups with the BT patients occupying an intermediate position. These results are reminiscent of the reports demonstrating under-expression of the *FCN3* gene in other carcinomas [32, 33]. However, estimation of the expression of either *FCN2* or *FCN3* gene does not distinguish between patients with less or more advanced disease stage, particular histological types or tumour grade, at least with the relatively small number of samples investigated here.

The combination of relatively elevated serum concentration (increased hepatic synthesis?) and low ovarian expression of both ficolin-2 and ficolin-3 seems to be a characteristic of ovarian malignancy. Indeed, *FCN3* gene expression inversely correlated with serum protein concentration in the OC group. Question whether these characteristic ficolin patterns (especially high serum levels, probably combined with other biomarkers) may in the future prove to be of value in screening asymptomatic women undoubtedly requires further investigation. Early identification of OC in apparently healthy women would be expected to lead to reduced mortality from the disease.

It has been reported that serum ficolins show age-related changes in concentration during childhood and differ from adult values [40]. In this study, ficolin-3 correlated with age. However, in contrast to the control group, there was no decline in serum ficolin-3 in OC patients after 64 years of age. Although correlation between ficolin-3 and ficolin-2 has been noted before [14, 30, 34] and confirmed here, no correlation of ficolin-2 with age was found.

In conclusion, the characteristic high systemic/low local ratio of ficolin-2 and ficolin-3 in ovarian and possibly other carcinomas warrants further investigation to explain these associations. In principle, complement-activating proteins might contribute not only to cancer progression but could also influence the course of underlying or accompanying diseases. For example, ficolin-2 can interact with β-1,3 glucan [41], and β-1,3 glucans are being investigated as potential anti-cancer therapy agents (reviewed by Vetvicka [42]). The physiological role of ficolins in the ovary and the reason for their diminished expression in malignant tissue needs to be clarified.

### Electronic supplementary material

Below is the link to the electronic supplementary material.

Supplementary material 1 (PDF 365 kb)

## Abbreviations

C: Controls
NO: Normal ovaries
BT: Benign tumours
OC: Ovarian cancer

## References

[1] Kilpatrick DC, Chalmers JD (2012). Human L-ficolin (ficolin-2) and its clinical significance. J Biomed Biotechnol.

[2] Tsujimura M, Ishida C, Sagara Y, Miyazaki T, Murakami K, Shiraki H, Okochi K, Maeda Y (2001). Detection of serum thermolabile beta-2 macroglycoprotein (Hakata antigen) by enzyme-linked immunosorbent assay using polysaccharide produced by *Aerococcus viridans*. Clin Diagn Lab Immunol.

[3] Swierzko A, Lukasiewicz J, Cedzynski M, Maciejewska A, Jachymek W, Niedziela T, Matsushita M, Lugowski C (2012). New functional ligands for ficolin-3 among lipopolysaccharides of *Hafnia alvei*. Glycobiology.

[4] Kuraya M, Ming Z, Liu X, Matsushita M, Fujita T (2005). Specific binding of L-ficolin and H-ficolin to apoptotic cells leads to complement activation. Immunobiology.

[5] Honore C, Hummelshoj T, Hansen BE, Madsen HO, Eggleton P, Garred P (2007). The innate immune component ficolin 3 (Hakata antigen) mediates the clearance of late apoptotic cells. Arthritis Rheum.

[6] Hummelshoj T, Munthe-Fog L, Madsen HO, Fujita T, Matsushita M, Garred P (2005). Polymorphisms in the *FCN2* gene determine serum variation and function of Ficolin-2. Hum Mol Genet.

[7] Hummelshoj T, Munthe-Fog L, Madsen HO, Garred P (2008). Functional SNPs in the human ficolin (*FCN*) genes reveal distinct geographical patterns. Mol Immunol.

[8] Herpers BL, Immink MM, de Jong BA, van Velzen-Blad H, de Jongh BM, van Hannen EJ (2006). Coding and non-coding polymorphisms in the lectin pathway activator L-ficolin gene in 188 Dutch blood bank donors. Mol Immunol.

[9] Cedzynski M, Nuytinck L, Atkinson AP, St Swierzko A, Zeman K, Szemraj J, Szala A, Turner ML, Kilpatrick DC (2007). Extremes of L-ficolin concentration in children with recurrent infections are associated with single nucleotide polymorphisms in the *FCN2* gene. Clin Exp Immunol.

[10] Garred P, Honore C, Ma YJ, Rorvig S, Cowland J, Borregaard N, Hummelshoj T (2010). The genetics of ficolins. J Innate Immun.

[11] Munthe-Fog L, Hummelshoj T, Ma YJ, Hansen BE, Koch C, Madsen HO, Skjodt K, Garred P (2008). Characterization of a polymorphism in the coding sequence of *FCN3* resulting in a ficolin-3 (Hakata antigen) deficiency state. Mol Immunol.

[12] Munthe-Fog L, Hummelshoj T, Honore C, Madsen HO, Permin H, Garred P (2009). Immunodeficiency associated with *FCN3* mutation and ficolin-3 deficiency. N Engl J Med.

[13] Schlapbach LJ, Thiel S, Kessler U, Ammann RA, Aebi C, Jensenius JC (2011). Congenital H-ficolin deficiency in premature infants with severe necrotising enterocolitis. Gut.

[14] Michalski M, Szala A, Swierzko St A, Lukasiewicz J, Maciejewska A, Kilpatrick DC, Matsushita M, Domzalska-Popadiuk I, Borkowska-Klos M, Sokolowska A, Szczapa J, Lugowski C, Cedzynski M (2012). H-ficolin (ficolin-3) concentrations and *FCN3* gene polymorphism in neonates. Immunobiology.

[15] Andersen JD, Boylan KL, Xue FS, Anderson LB, Witthuhn BA, Markowski TW, Higgins L, Skubitz AP (2010). Identification of candidate biomarkers in ovarian cancer serum by depletion of highly abundant proteins and differential in-gel electrophoresis. Electrophoresis.

[16] Hummelshoj T, Munthe Fog L, Madsen HO, Sim RB, Garred P (2008). Comparative study of the human ficolins reveals unique features of ficolin-3 (Hakata antigen). Mol Immunol.

[17] Kuraya M, Matsushita M, Endo Y, Thiel S, Fujita T (2003). Expression of H-ficolin/Hakata antigen, mannose-binding lectin-associated serine protease (MASP)-1 and MASP-3 by human glioma cell line T98G. Int Immunol.

[18] Swierzko AS, Florczak K, Cedzynski M, Szemraj J, Wydra D, Bak-Romaniszyn L, Emerich J, Sulowska Z (2007). Mannan-binding lectin (MBL) in women with tumours of the reproductive system. Cancer Immunol Immunother.

[19] Leth-Larsen R, Floridon C, Nielsen O, Holmskov U (2004). Surfactant protein D in the female genital tract. Mol Hum Reprod.

[20] Hansen S, Selman L, Palaniyar N, Ziegler K, Brandt J, Kliem A, Jonasson M, Skjoedt MO, Nielsen O, Hartshorn K, Jørgensen TJ, Skjødt K, Holmskov U (2010). Collectin 11 (CL-11, CL-K1) is a MASP-1/3-associated plasma collectin with microbial-binding activity. J Immunol.

[21] Kilpatrick DC, Fujita T, Matsushita M (1999). P35, an opsonic lectin of the ficolin family, in human blood from neonates, normal adults, and recurrent miscarriage patients. Immunol Lett.

[22] Szala A, Swierzko St A, Cedzynski M (2013). Cost-effective procedures for genotyping of human *FCN2* gene single nucleotide polymorphisms. Immunogenetics.

[23] Li YL, Ye F, Hu Y, Lu WG, Xie X (2009). Identification of suitable reference genes for gene expression studies of human serous ovarian cancer by real-time polymerase chain reaction. Anal Biochem.

[24] Winer J, Jung CK, Shackel I, Williams PM (1999). Development and validation of real-time quantitative reverse transcriptase-polymerase chain reaction for monitoring gene expression in cardiac myocytes in vitro. Anal Biochem.

[25] Bertenshaw GP, Yip P, Seshaiah P, Zhao J, Chen T-H, Wiggins WS, Mapes JP, Mansfield BC (2008). Multianalyte profiling of serum antigens and autoimmune and infectious disease molecule to identify biomarkers dysregulated in epithelial ovarian cancer. Cancer Epidemiol Biomarkers.

[26] Ytting H, Jensenius JC, Christensen IJ, Thiel S, Nielsen HJ (2004). Increased activity of the mannan-binding lectin complement activation pathway in patients with colorectal cancer. Scand J Gastroenterol.

[27] Ytting H, Christensen IJ, Thiel S, Jensenius JC, Nielsen HJ (2005). Serum mannan-binding lectin-associated serine protease 2 levels in colorectal cancer: relation to recurrence and mortality. Clin Cancer Res.

[28] Vazquez-Ortíz G, Ciudad CJ, Piña P, Vazquez K, Hidalgo A, Alatorre B, Garcia JA, Salamanca F, Peralta-Rodriguez R, Rangel A, Salcedo M (2005). Gene identification by cDNA arrays in HPV-positive cervical cancer. Arch Med Res.

[29] Arellano-Garcia ME, Li R, Liu X, Xie Y, Yan X, Lod JA, Hu S (2010). Indentification of tetranectin as a potential biomarker for metastatic oral cancer. Int J Mol Sci.

[30] Kilpatrick DC, McLintock LA, Allan EK, Copland M, Fujita T, Jordanides NE, Koch C, Matsushita M, Shiraki H, Stewart K, Tsujimura M, Turner ML, Franklin IM (2003). No strong relationship between mannan binding lectin or plasma ficolins and chemotherapy-related infections. Clin Exp Immunol.

[31] Ameye L, Paesmans M, Thiel S, Jensenius JC, Aoun M (2012). M-ficolin levels are associated with the occurrence of severe infections in patients with haematological cancer undergoing chemotherapy. Clin Exp Immunol.

[32] Luo JH, Ren B, Keryanov S, Tseng GC, Rao UN, Monga SP, Strom S, Demetris AJ, Nalesnik M, Yu YP, Ranganathan S, Michalopoulos GK (2006). Transcriptomic and genomic analysis of human hepatocellular carcinomas and hepatoblastomas. Hepatology.

[33] Shi I, Hashemi Sadraei N, Duan ZH, Shi T (2011). Aberrant signaling pathways in squamous cell lung carcinoma. Cancer Inform.

[34] Schlapbach LJ, Aebi C, Hansen AG, Hirt A, Jensenius JC, Ammann RA (2009). H-ficolin serum concentration and susceptibility to fever and neutropenia in paediatric cancer patients. Clin Exp Immunol.

[35] Hefler-Frischmuth K, Hefler LA, Heinze G, Paseka V, Grimm C, Tempfer CB (2009). Serum C-reactive protein in the differential diagnosis of ovarian masses. Eur J Obstet, Gynecol Reprod Biol.

[36] Andersen T, Munthe-Fog L, Garred P, Jacobsen S (2009). Serum levels of ficolin-3 (Hakata antigen) in patients with systemic lupus erythematosus. J Rheumatol.

[37] Liu J, Ali MA, Shi Y, Zhao Y, Luo F, Yu J, Xiang T, Tang J, Li D, Hu Q, Ho W, Zhang X (2009). Specifically binding of L-ficolin to N-glycans of HCV envelope glycoproteins E1 and E2 leads to complement activation. Cell Mol Immunol.

[38] Hoang TV, Toan NL, le Song H, Ouf EA, Bock CT, Kremsner PG, Kun JF, Velavan TP (2011). Ficolin-2 levels and *FCN2* haplotypes influence hepatitis B infection outcome in Vietnamese patients. PLoS ONE.

[39] Roy S, Biswas S, Saroha A, Sahu D, Das HR (2012). Enhanced expression and fucosylation of ficolin3 in plasma of RA patients. Clin Biochem.

[40] Sallenbach S, Thiel S, Aebi C, Otth M, Bigler S, Jensenius JC, Schlapbach LJ, Ammann RA (2011). Serum concentrations of lectin-pathway components in healthy neonates, children and adults: mannan-binding lectin (MBL), M-, L-, and H-ficolin, and MBL-associated serine protease-2 (MASP-2). Pediatr Allergy Immunol.

[41] Ma YG, Cho MY, Zhao M, Park JW, Matsushita M, Fujita T, Lee BL (2004). Human mannose-binding lectin and L-ficolin function as specific pattern recognition proteins in the lectin activation pathway of complement. J Biol Chem.

[42] Vetvicka V (2011). Glucan—immunostimulant, adjuvant, potential drug. World J Clin Oncol.

