# Supplementary material for: Ficolin-2 and ficolin-3 in women with malignant and benign ovarian tumours
Source: Cancer Immunol Immunother. 2013 Jun 7;62(8):1411–9. doi: 10.1007/s00262-013-1445-3 (PMC3717161; doi:10.1007/s00262-013-1445-3)
Supplement: Supplementary file 1 — Supplementary material 1 (PDF 365 kb) [file 262_2013_1445_MOESM1_ESM.pdf]

Supplementary material:

Ficolins-2 and -3 in women with malignant and benign ovarian tumours

Agnieszka Szala, Sambor Sawicki, Anna St. Swierzko, Janusz Szemraj, Marcin Sniadecki, Mateusz Michalski, Andrzej Kaluzynski, Jolanta Lukasiewicz, Anna Maciejewska, Dariusz Wydra, David C. Kilpatrick, Misao Matsushita, Maciej Cedzynski

Correspondence to: Maciej Cedzynski, Laboratory of Immunobiology of Infections, Institute of Medical Biology, Polish Academy of Sciences, Lodowa 106, 93-232 Lodz, Poland; e-mail: mcedzynski@cbm.pan.pl

Table S1. Primers used for investigation of -64 A/C (rs7865453) *FCN2* gene polymorphism. Substitution of two nucleotides in P64_RA primer (underlined) was introduced to enhance its specificity (according to Szala et al., 2013 [22]).

**Primer**	**5'-3' sequence**
P64_F	CACAAGCAAGTCAGCCTGTT
P64_Ctrl	CAGCTTTCAGGGACGAGAAG
P64_RA	GGCTAGAGAAGCCAGCCT<u>TA</u>CT
P64_RC	GGCTAGAGAAGCCAGCCTCCCG

Table S2. Primers used for investigation of -4 A/G (rs17514136) *FCN2* gene polymorphism (according to Szala et al., 2013 [22]).

**Primer**	**5'-3' sequence**
P4_F	GAGCAGCCCTGGAGATGAT
P4_R	AGAAGTTTCCAGGAGGAGGC

Table S3. Primers used for investigation of +6359 C/T (rs17549193) *FCN2* gene polymorphism. Substitutions of nucleotides (underlined) were introduced enhance the specificity of P6359_F2 and P6359_R2 primers (according to Szala et al., 2013 [22]).

Primer	Primer sequence 5'-3'
P6359_F1	TTGCACTTCTTGGATTGTGC
P6359_F2	CCTGCACAGGAGATTCCCGGAT
P6359_R1	GGACTGGTTGTTGTTGAACG
P6359_R2	TGGCAGTTTTTGTACCACCA

Table S4. Primers used for investigation of +6424 G>T (rs7851696) *FCN2* gene polymorphism. Substitutions of nucleotides (underlined) were introduced to enhance the specificity of P6424_FG and P6424_FT primers (according to Szala et al., 2013 [22]).

Primer	Primer sequence 5'-3'
P6424_FG	GATCTTAACACCGGAAATGGTG
P6424_FT	GATCTTAACACCGGAAATAGTT
P6424_Rev	TTACAAACCGTAGGGCCAAG
P6424_Ctrl	ACGATGCTCACATTTCCTCC

Table S5. Primers used for investigation of *1637delC* (rs28357092) *FCN3* gene frame-shift mutation (according to Michalski et al., 2012 [14])

Primer	Primer sequence 5'-3'
Forward	GATCACATGGTGTTGGGTGT
Reverse	TTCACTCTTTTCACCCAGGC

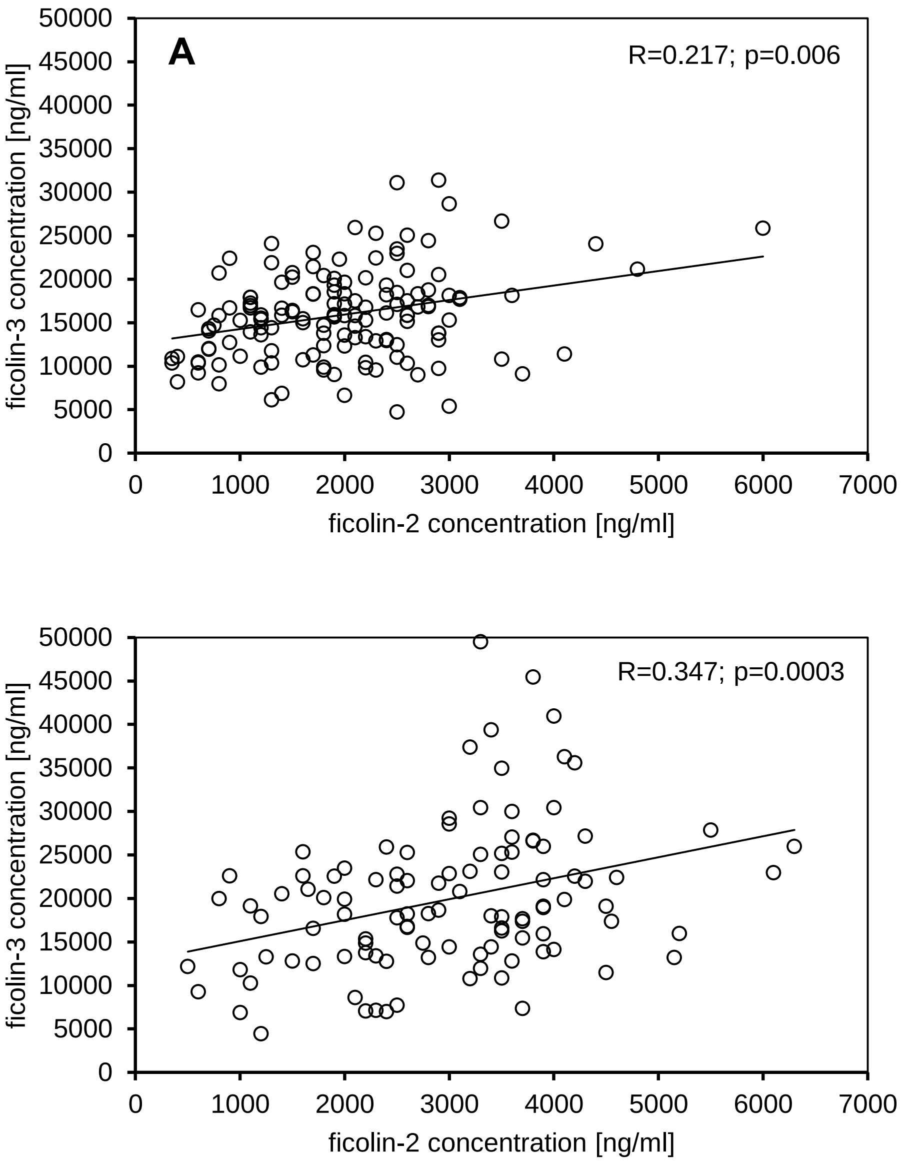

Fig. S1. Correlation between serum concentrations of ficolin-2 and ficolin-3 within the control (**A**) and ovarian cancer (**B**) groups.

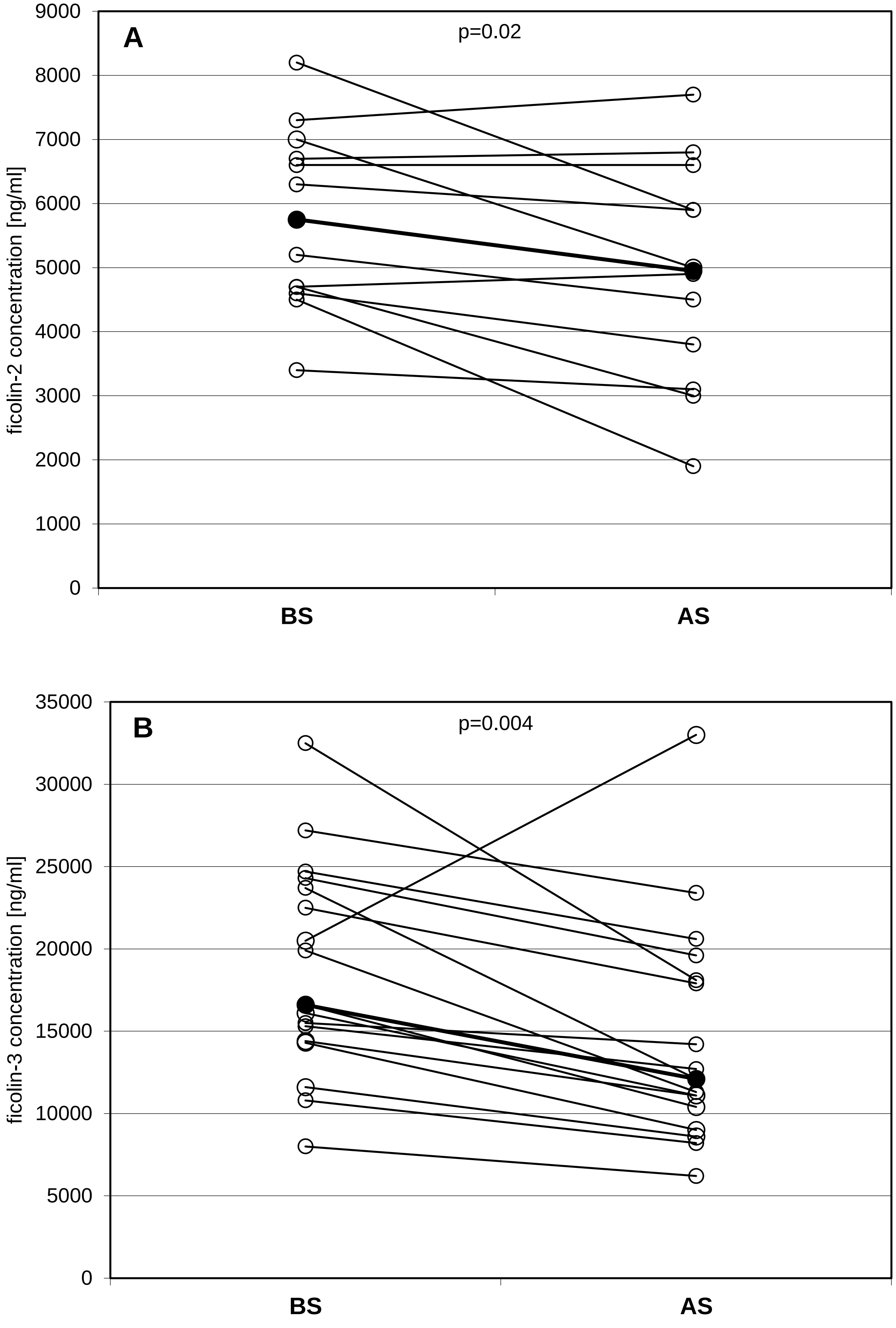

Fig. S2. Pre- (BS) and post-operative (AS) serum concentrations of ficolin-2 (n=12) (**A**) and ficolin-3 (n=17) (**B**), in ovarian cancer patients. Filled symbols indicate medians.

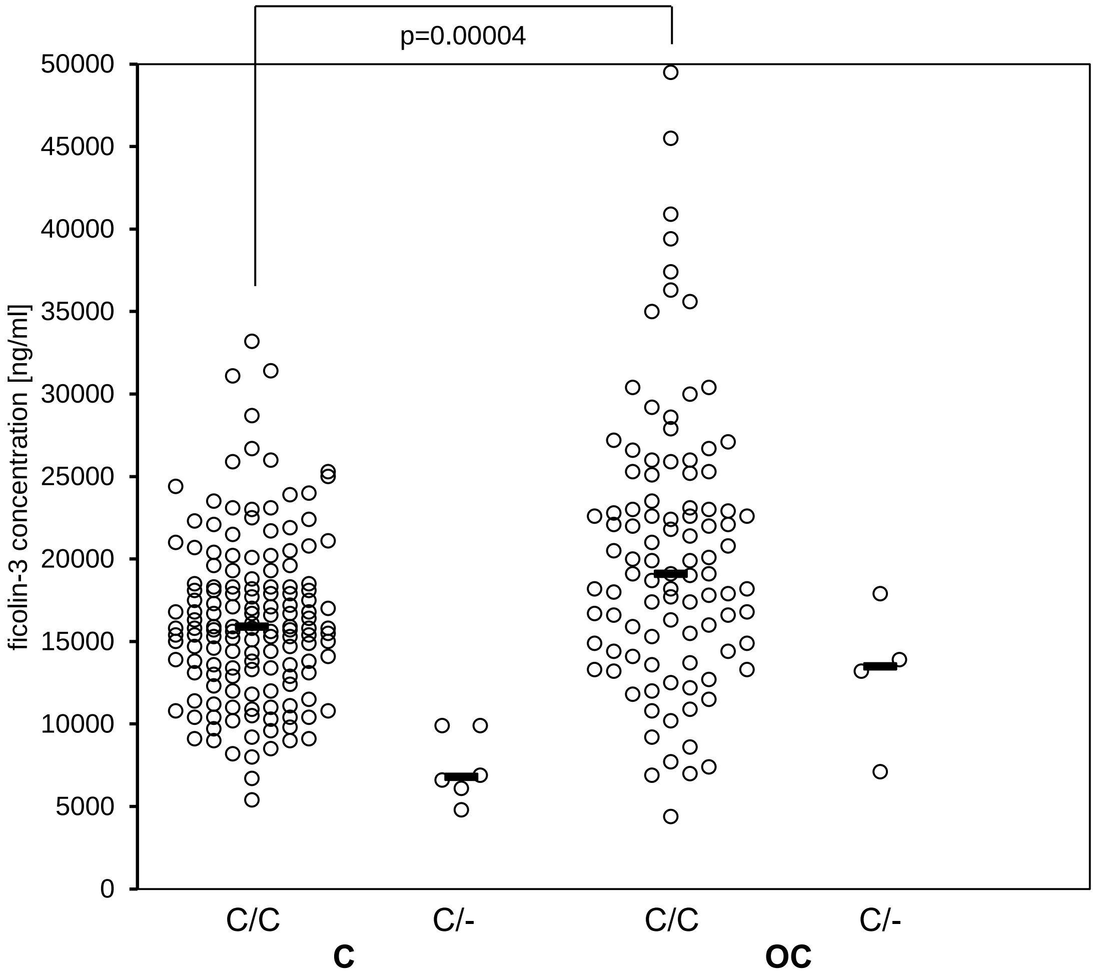

Fig. S3. Individual concentrations of ficolin-3, depending on *FCN3* genotype within the control (C) and ovarian cancer (OC) groups. Bars indicate median values.

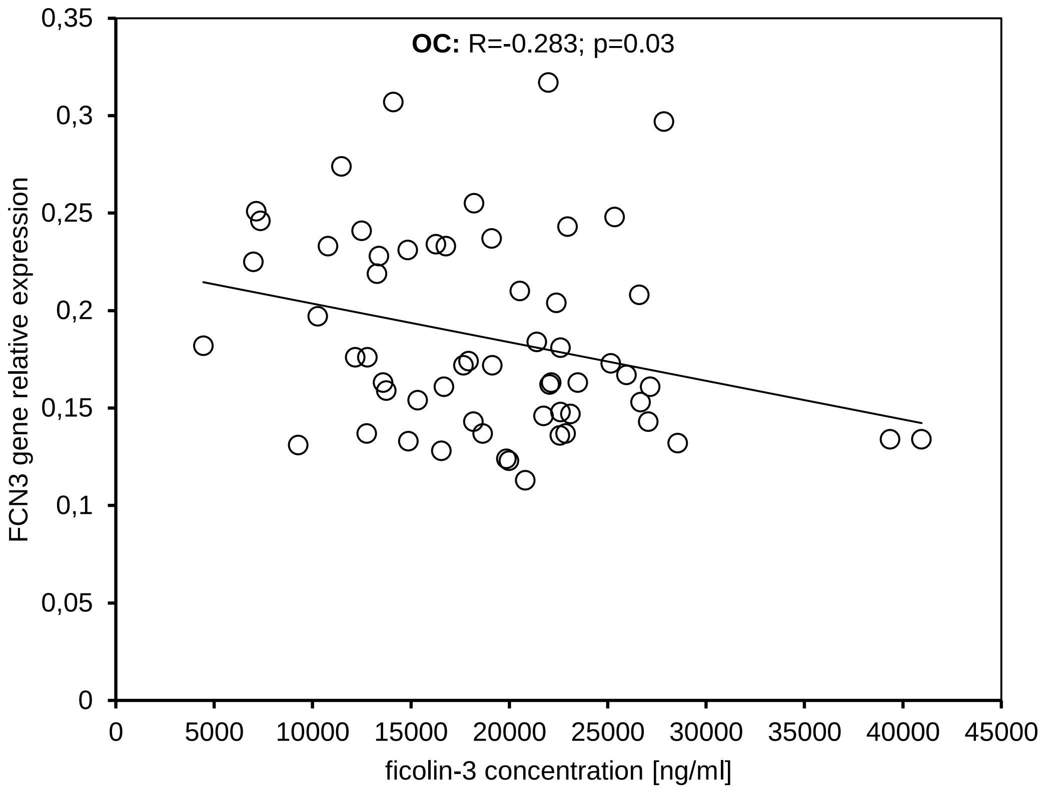

Fig. S4. Inverse correlation between *FCN3* gene expression and ficolin-3 levels among ovarian cancer patients.
